# Do COVID-19-Related Stress, Being Overweight, and Body Dissatisfaction Contribute to More Disordered Eating in Polish Women?—A Cluster Analysis Approach

**DOI:** 10.3390/ijerph182413100

**Published:** 2021-12-12

**Authors:** Kamila Czepczor-Bernat, Justyna Modrzejewska, Adriana Modrzejewska, Paweł Matusik

**Affiliations:** 1 Institute of Psychology, University of Wrocław, 50-527 Wrocław, Poland; 2 Institute of Pedagogy, University of Bielsko-Biala, 43-309 Bielsko-Biala, Poland; jmodrzejewska@ath.bielsko.pl; 3 Department of Psychology, School of Health Sciences in Katowice, Medical University of Silesia in Katowice, 40-055 Katowice, Poland; adriana.modrzejewska@sum.edu.pl; 4 Department of Pediatrics, Pediatric Obesity and Metabolic Bone Diseases, Faculty of Medical Sciences in Katowice, Medical University of Silesia, 40-055 Katowice, Poland; pmatusik73@gmail.com

**Keywords:** COVID-19-related stress, overweight, body dissatisfaction, disordered eating, women

## Abstract

We hypothesized that women who are overweight, experiencing COVID-19-related stress, and with high body dissatisfaction would have significantly greater disordered eating than those of healthy weight, without stress, and with low body dissatisfaction. Participants (*N* = 1354 women; *M*_age_= 31.89 years, *SD* = 11.14) filled in the Contour Drawing Rating Scale, the Emotional Overeating Questionnaire, the Eating Motivation Survey, the Mindful Eating Questionnaire, and a COVID-19-related stress measure and sociodemographic survey. The cluster analysis technique revealed four distinct clusters: (a) Cluster 1 (*N* = 314): healthy body weight, no COVID-related stress, and low body dissatisfaction (*M* = 1.19); (b) Cluster 2 (*N* = 131): overweight, no COVID-related stress, and high body dissatisfaction (*M* = 2.41); (c) Cluster 3 (*N* = 597): healthy body weight, COVID-related stress, and low body dissatisfaction (*M* = 1.27); (d) Cluster 4 (*N* = 312): overweight, COVID-related stress, and high body dissatisfaction (*M* = 2.84). Generally, our outcomes partially support our hypothesis, as higher levels of some types of disordered eating were observed in women who were overweight with COVID-related stress and high body dissatisfaction (Cluster 4) as compared with women with healthy body weight, no COVID-related stress, and with low levels of body dissatisfaction (Cluster 1). Our results indicate that both body weight status, as well as COVID-19-related stress and body dissatisfaction, may contribute to the intensity of disordered eating. During future epidemic-related quarantines, this may be an argument in favor of organizing support regarding emotional functioning, body image, and eating behaviors, particularly for the most vulnerable groups—including overweight and obese women.

## 1. Introduction

There have been a lot of data published recently on the impact of the coronavirus pandemic on health [1,2,3]. The situation has given rise to concerns for one’s own health [4,5], and many reports show how COVID-19 and restrictions related to it can affect both psyche and soma [2,6,7,8,9,10,11]. Within this context, research findings have also been published on, inter alia: (a) changes in eating behavior [12,13,14]; (b) the relationships between COVID-19-related stress, disordered eating (e.g., emotional overeating) [15,16,17], symptoms of eating disorders, and negative body image [18,19,20,21,22]; (c) the functioning of people who are overweight and have possible changes in body weight [13,23].

The results so far indicate that for many people, COVID-19 was (or still is) a strong source of stress [4,22,24], and its high levels can lead to unhealthy eating behaviors [16,17,25,26]. These changes in eating were (and may still be) related to the fact that people use food as a way of regulating emotions, especially in a situation where other ways of coping with anxiety, stress, and other negative emotional states have been significantly limited by restrictions related to COVID-19 (including direct contact with friends, going to the cinema and other entertainment venues, etc.) [15,16,24,25,27,28]. In addition, the source of negative emotions may also be COVID-related restrictions, which, at some stage of the pandemic, prevented us from practicing physical activity outdoors, in the gym, and in other places of recreation [8,13]. The COVID-19 situation may therefore be responsible for the weight-loss and maintenance strategies that have characterized the main changes in eating behavior. These may have led to the appearance or intensification of a feeling of lack of weight control and exacerbated perceived stress, and, as a result, led people to try to cope with these emotions in a maladaptive way (e.g., through emotional overeating) [13,15,16,20,25,28,29,30,31]. Moreover, previous research (conducted before COVID-19) indicated that the group of the greatest risk for the development of disturbed eating behaviors could be the group of people who are overweight, who are at the same time strongly dissatisfied with their body [32,33]. It is therefore worth asking whether and how, at the same time, these factors—COVID-19-related stress, being overweight, and body dissatisfaction—can have contributed to more disordered eating during the COVID-19 pandemic?

Referring to the category of disordered eating, it should be mentioned that this is a very broad category including, among others, emotional (over)eating and a low level of mindful eating. Emotional (over)eating can be defined as the tendency to regulate emotional states by eating (too much) food [15,34,35]. In turn, a low level of mindful eating is associated with (a) a high level of the diet mentality (a rigid tendency to make food choices without taking account of fullness or hunger, as well as daily life circumstances and individual preferences because the priority is body weight management); (b) a low level of the non-judgmental process of paying attention to the eating experience by listening to and having an awareness of physical hunger and satiety cues, (c) a low level of eating by engaging all of the senses and only until we are full [36]. Low levels of mindful eating and high levels of emotional eating will significantly increase the risk of weight gain [28,37], which may result in an intensification of body dissatisfaction [33].

When writing about eating behaviors, it should also be mentioned that we can evaluate them by examining the motivation underlying particular food choices [38]. Referring to the situation related to COVID-19 and to the above findings, it would be worth looking at such motives of eating such as health, weight control, and affecting regulation [4,5,15,17,25,28].

To sum up, although knowledge about COVID-19-related stress, body weight, and eating behaviors/disorders has increased in diverse populations [12,13,22,25], knowledge and research on the importance of body dissatisfaction to disordered eating during COVID-19 remains limited. Given the likelihood that during COVID-19, in the UK, women have reported (compared to men) a stronger worsening in unhealthy eating and negative body image [39], and, taking into account previous findings (prior to COVID-19) that indicate a particularly high level of body dissatisfaction among women who are overweight [33], greater knowledge and further research is vital for a fuller understanding of this issue in women (also in the Polish sample). Therefore, our study aimed to (1) classify different conditions associated with weight status, COVID-19-related stress, and body dissatisfaction; and (2) analyze and compare disordered eating in women with different weight status, COVID-19-related stress, and body dissatisfaction. We hypothesized that women who were overweight, experiencing COVID-19-related stress, and with high body dissatisfaction, would have significantly greater disordered eating than those of healthy weight, without stress, and with low body dissatisfaction.

## 2. Materials and Methods

### 2.1. Participants and Procedure

Our cross-sectional study was approved by the University Ethics Committee (no. 2021/2/2E/2) and conducted in accordance with the international standards of research ethics (Helsinki Declaration, 2001). From January to March 2021, Polish women were recruited via an online advertisement (e.g., on social media networks) and flyers (posted at workplace locations, universities, etc.). After contacting the researchers, all women were made aware that participation in this study was anonymous and voluntary. Next, they were asked to read informed consent to participate in the study. After becoming acquainted with all the information, if they expressed their willingness to participate in the study, they signed this informed consent online. Next, women completed an online survey (populated on Google Forms), which was accessed via a link sent to participants. Participants were not remunerated.

One thousand five hundred and ninety-four people volunteered for the study. Two hundred and forty participants had significant missing data gaps or were outliers (due to the aim of this analysis—assessment in the group of women who were healthy or overweight—men and all those who were not overweight were removed from the database). Finally, our sample included 1354 Polish women who ranged in age from 18 to 72 years (*M* = 31.89, *SD* = 11.14).

### 2.2. Measures

#### 2.2.1. The Contour Drawing Rating Scale (CDRS)

To assess body dissatisfaction, participants completed the Contour Drawing Rating Scale [40]. The CDRS consists of nine consecutively numbered female silhouettes. Participants were asked (a) “Which silhouette reflects what you look like now?” (“real silhouette”); (b) “Which silhouette reflects what you would like to look like?” (“ideal silhouette”). The level of body dissatisfaction was assessed by subtracting the number of the silhouette indicated as “real” from the number of the silhouette indicated as “ideal“, and the higher the discrepancy between them, the greater the body dissatisfaction.

#### 2.2.2. The Emotional Overeating Questionnaire (EOQ)

To assess emotional overeating, participants completed the Emotional Overeating Questionnaire [41]. The nine-item EOQ measures how often people have eaten excessive amounts of food when they have felt an emotional state (anger, sadness, loneliness, anxiety, tiredness, boredom, guilt, happiness) and physical pain over the last 28 days. All items were rated on a 7-point scale ranging from 0 (“no days”) to 6 (“everyday”). The higher the score, the higher the level of emotional overeating. To obtain a Polish translation, we used a standard forward–backward translation procedure. In our study, Cronbach’s alpha coefficient was acceptable (α = 0.91).

#### 2.2.3. The Eating Motivation Survey (EMS)

To assess how often people eat for health reasons, to control body weight, and to regulate emotion, participants completed the Eating Motivation Survey [38]. The 45-item EMS includes the following 15 subscales measuring different eating motives: habits, need and hunger, liking, health, pleasure, traditional eating, convenience, natural concerns, price, visual appeal, sociability, weight control, affect regulation, social image, social norms. Due to the aim of the study, only three subscales were selected for analysis—health, weight control, affect regulation. All items were rated on a 7-point scale ranging from 1 (“never”) to 7 (“always”). The higher the score, the more strongly eating is determined by health, weight control, and affect regulation. To obtain a Polish translation, we used a standard forward–backward translation procedure. In our study, Cronbach’s alpha coefficient was acceptable (α_health_ = 0.82, α_weightcontrol_ = 0.81, α_affectregulation_ = 0.80).

#### 2.2.4. The Mindful Eating Questionnaire (MEQ)

To assess mindful eating, participants completed the Mindful Eating Questionnaire [42]. The 20-item EMS includes two subscales: (a) recognition—eating is conditioned by hunger and satiety, (b) awareness—eating while being aware of physiological and psychological experiences. All items were rated on a 4-point scale ranging from 1 (“never/rarely”) to 4 (“usually/always”). The higher the score, the more mindfully people eat. To obtain a Polish translation, we used a standard forward–backward translation procedure. In our study, Cronbach’s alpha coefficient was acceptable (α_recognition_ = 0.70, α_awareness_ = 0.82).

#### 2.2.5. COVID-19-Related Stress

To assess COVID-19-related stress, participants were asked “Is the current epidemiological situation related to COVID-19 stressful for you?” and they could choose to respond: “No” (0) or “Yes” (1) (a dummy-coded variable).

#### 2.2.6. Sociodemographic Variables

Participants were asked about gender, weight, height, age, sexual orientation, and ethnicity. Body mass index (BMI; kg/m^2^) was calculated based on self-reported weight and height. Based on the World Health Organization guidelines [43], women were divided into two groups: (a) healthy body weight: 18.5 ≤ BMI ≤ 24.99 kg/m^2^, and (b) overweight (pre-obesity and obesity): BMI ≥ 25 kg/m^2^.

### 2.3. Statistical Analysis

A two-step cluster analysis (with Schwarz’s Bayesian criterion) was chosen to identify clusters based on BMI, COVID-related stress, and body dissatisfaction. This analysis is appropriate for samples larger than 200 and for both categorical and continuous variables [44]. One-way analysis of variance (ANOVA) with Bonferroni multiple comparison tests was used to assess differences between the clusters with regard to emotional overeating, eating motives (health, weight control, affect regulation), and mindful eating (recognition, awareness). The significance level was defined as *p*  <  0.05. All analyses were conducted using IBM SPSS Statistic version 26 (IBM, Armonk, New York, United States).

## 3. Results

### 3.1. Cluster Analysis of Weight Status (BMI), COVID-19-Related Stress, and Body Dissatisfaction

The analysis included 1354 Polish women who ranged in: (a) body weight from 45 to 130 kg (*M* = 67.02, *SD* = 13.04), (b) height from 146 to 188 cm (*M* = 166.22, *SD* = 6.09), and (c) body mass index (BMI) from 18.50 to 47.96 kg/m^2^ (*M* = 24.23 *SD* = 4.40). Nine hundred and eleven women (67.28%) had normal body weight, and 443 women (32.72%) were overweight.

The cluster analysis technique revealed four distinct clusters (Table 1): (a) Cluster 1 (*N* = 314): healthy body weight, no COVID-related stress, and low body dissatisfaction (*M* = 1.19); (b) Cluster 2 (*N* = 131): overweight, no COVID-related stress, and high body dissatisfaction (*M* = 2.41); (c) Cluster 3 (*N* = 597): healthy body weight, COVID-related stress, and low body dissatisfaction (*M* = 1.27); (d) Cluster 4 (*N* = 312): overweight, COVID-related stress, and high body dissatisfaction (*M* = 2.84).

### 3.2. Comparison of the Four Clusters for Emotional Overeating, Eating Motives, and Mindful Eating

One-way analysis of variance (ANOVA) showed the significant effect of clusters on emotional overeating, two eating motives (health, affect regulation), and mindful eating (recognition, awareness). Table 1 shows the results of multiple comparisons.

Referring to the most important findings, our results indicated that Cluster 4 (overweight, COVID-related stress, high body dissatisfaction) had significantly greater disordered eating than Clusters 1 (healthy body weight, no COVID-related stress, low body dissatisfaction) and 3 (healthy body weight, COVID-related stress, low body dissatisfaction) and these significant differences were found with regard to emotional overeating, health motive, and affecting regulation motive of eating, and to one aspect of mindful eating—recognition. In turn, for the second aspect of mindful eating—awareness—the differences between Cluster 4 and Cluster 3 were at *p* = 0.076.

Cluster 4 differed significantly from Cluster 2 (overweight, no COVID-related stress, high body dissatisfaction) in terms of emotional overeating and affect regulation motive for eating. Moreover, Cluster 1 also had a lower level of emotional overeating and affect regulation motive of eating than Cluster 3, and Cluster 3 had significantly higher health motive for eating and awareness than Cluster 2. Interestingly, in relation to weight control motive for eating, we did not find any significant differences between Clusters 1, 2, 3, and 4, and Clusters 1 and 2 did not differ in terms of any of the analyzed variables.

Generally, our outcomes partially support our hypothesis, as higher levels of some types of disordered eating were observed in women who were overweight, with COVID-related stress and high body dissatisfaction as compared with women with healthy body weight, no COVID-related stress, and with low levels of body dissatisfaction. Our results indicate that body weight status, as well as COVID-19-related stress and body dissatisfaction, may contribute to the intensity of disordered eating.

## 4. Discussion

Our study examined patterns of weight status, COVID-19-related stress, and body dissatisfaction in a sample of Polish women during the COVID-19 pandemic. Four clusters were discovered. By analyzing the most important findings, significant differences (in terms of most of the assessed behaviors and eating motives) were found between women who were overweight who were simultaneously stressed by COVID-19 and had a high level of body dissatisfaction (Cluster 4), and women with a healthy body weight, who experienced no stress from COVID-19 and had a low level of body dissatisfaction (Cluster 1). Women from Cluster 4 (compared to Cluster 1) significantly more often declared that their motive for undertaking eating behavior during COVID-19 was the regulation of emotions (a subscale of the Eating Motivation Survey [38]). This tendency is also reflected in the outcomes of emotional overeating (as measured by the Emotional Overeating Questionnaire [41])—women from Cluster 4 overeat more often under the influence of various emotional states and physical pain compared to Cluster 1. Moreover, the motive for undertaking the eating behaviour of Cluster 4 was significantly less often health, and less often that they finished their meals in response to the feeling of satiety (i.e., they more often continued their meal as a form of overeating despite the fact that they were already full; mindful eating—“recognition”). The above-described pattern of disordered eating of Cluster 4 may be particularly risky, as its consolidation may result in further weight gain and/or difficulties in effective weight reduction [34,45]. Therefore, taking into account not only emotional factors (such as stress) but also weight status and body dissatisfaction will help to better understand the mechanisms by which abnormal eating behaviour develops [36,46]. These outcomes suggest it may be of critical importance during COVID-19 to promote a healthy body image and improve emotional regulation for women who are overweight.

The importance of COVID-19-related stress for more disordered eating in a sample of Polish women may also be indicated by some of our other results. This is evidenced by significantly higher results in affect regulation motive of eating and emotional overeating in Cluster 4 compared to Cluster 2 (overweight, no COVID-related stress, high body dissatisfaction), and similar differences between Clusters 1 (healthy body weight, no COVID-related stress, low body dissatisfaction) and 3 (healthy body weight, COVID-related stress, low body dissatisfaction). Thus, both when comparing clusters composed of overweight women and women with a healthy body weight, emotional (over)eating is stronger in groups that are stressed by COVID-19. This may mean that although this tendency among women with a healthy body weight is not as strong as among those who are overweight [34,45], it is also present in the former group and may be intensified as a result of experiencing tension from COVID-19 and related restrictions. Both groups should therefore be subjected to influences/activities that will help the woman regulate her emotions in a more adaptive way than through emotional (over)eating [15,16,25,28,31].

When planning some interventions, there are other important aspects to consider—body image and body weight. In the context of our research, it turns out that if women did not accept their silhouettes (and the related body weight), then their eating behaviors were more disordered. This is evidenced by the differences described above between Cluster 4 and Cluster 1, as well as the differences between Clusters 4 and 3 and between Clusters 2 and 3. The differences between Clusters 3 and 4 show that women who are overweight, with COVID-19-related stress and high body dissatisfaction (compared to Cluster 3: healthy body weight, COVID-related stress, low body dissatisfaction) were more likely to eat under the influence of emotions and physical pain, less often likely to eat taking into account health and less often likely to eat mindfully (mindful eating subscales: “recognition” *p* < 0.01 and “awareness” *p* = 0.076). This could mean that even though both groups (Clusters 3 and 4) are stressed by COVID-19, more disordered eating patterns have emerged in women who are overweight and have high levels of body dissatisfaction. Furthermore, even when we compared Clusters 2 and 3 (differing in their experience of COVID-19-related stress), it turned out that in the group with high body dissatisfaction, who were overweight and had no COVID-related stress (Cluster 2), we observed a more disturbed eating (lower health motive of eating and lower awareness) compared to the group with low body dissatisfaction, healthy body weight, and COVID-related stress (Cluster 3).

Interestingly, it was not possible to obtain any significant differences with regard to the subscale of the Eating Motivation Survey [38]—weight control. This means that women from all clusters (with a similar frequency) were motivated to manage their own body weight through making certain food choices during the COVID-19 period. A possible explanation of these results may be related to the fact that when the possibilities of practicing physical activity in the previous way (e.g., in the gym) were limited, diet management became one of the main methods of weight control in the groups of various weight status. However, more research is needed in this regard.

Overall, our results partially support our hypothesis, as higher levels of some types of disordered eating were observed in overweight women with COVID-related stress and high body dissatisfaction, compared with women with healthy body weight, no COVID-related stress, and with low levels of body dissatisfaction. These outcomes indicate that body weight status, as well as COVID-19-related stress and body dissatisfaction, may contribute to the intensity of disordered eating. Therefore, it is important to design interventions that target changes in emotional functioning, cognitive functioning, and body image in a sample of women. These activities should include, but are not limited to, (a) managing stress, emotions, and physical pain in a more adaptive way than by (over)eating; (b) learning to accept the body, taking into account negative emotional states such as stress and anxiety, which could be aggravated by COVID-19 and its limitations; (c) learning more adaptive forms of managing one’s own body weight than through disordered eating; (d) developing and consolidating a diet model in which one of the primary eating motives is health.

Our findings are important because understanding how COVID-19 experiences and people’s individual predisposition (associated with body shape and weight) differentially relate to eating behaviors has direct implications for tailoring prevention efforts and treatment. Our study shows that COVID-19-related lockdowns and related consequences (e.g., increasing stress and concerns about body shape and weight) may affect eating behaviors. During future epidemic-related quarantines, this may be an argument in favor of organizing support regarding emotional functioning, body image, and eating behaviors, particularly for the most vulnerable groups—including overweight and obese women. Taking such actions seems to be important in the light of reports which show that disordered eating can intensify over time and lead to the development of a full syndrome of eating disorders [47]. And, as recent research shows, the problem of eating disorders has increased significantly during the COVID-19 pandemic, which was also associated with a higher percentage of patients in this group having suicidal ideation and attempting suicide [48].

Our study also had some limitations: (a) study design: a cross-sectional study; (b) participants: only female volunteers; (c) measures: only self-reported data, one-item COVID-related stress measurement, Cronbach’s alpha for “recognition” = 0.70, and, with regard to some measures, there is no validation on the Polish sample. It is therefore necessary to plan further studies that take into account these limitations (e.g., longitudinal studies, women and men, and comparison of their functioning, objective measurement of body weight and height, and validated instruments for measuring of stress [49] and other variables). Moreover, note that it is also possible that if the clusters have weight differences, it is likely that they already had differences in eating habits, and perhaps differences in the levels of disordered eating, independently of other variables. Therefore, one should be careful in drawing conclusions, and this issue should be taken into account in future longitudinal studies. On a final note, it is necessary to emphasize that our results may have a limited possibility of generalization due to the specificity of the lockdown conditions in Poland.

## Figures and Tables

**Table 1:** Between-cluster differences: age, body mass index, emotional overeating, eating motives, and mindful eating.

	CLUSTER 1 (*N* = 314): Healthy Body Weight + No COVID-Related Stress + Low Body Dissatisfaction (CDRS)	CLUSTER 2 (*N* = 131): Overweight + No COVID-Related Stress + High Body Dissatisfaction (CDRS)	CLUSTER 3 (*N* = 597): Healthy Body Weight + COVID-Related Stress + Low Body Dissatisfaction (CDRS)	CLUSTER 4 (*N* = 312): Overweight + COVID-Related Stress + High Body Dissatisfaction (CDRS)	
	*M* (*SD*)				*Post hoc*
	*F*(3, 1350) = 45.01, *p* < 0.001, *ƞ_p_*^2^ = 0.09				
**Age**	29.41 (10.00)	36.08 (11.27)	29.63 (10.46)	36.95 (11.31)	1 vs. 2 *** 1 vs. 3 1 vs. 4 *** 2 vs. 3 *** 2 vs. 4 3 vs. 4 ***
	*F*(3, 1350) = 699.80, *p <* 0.001, *ƞ_p_*^2^ = 0.61				
**Body mass index**	21.91 (1.81)	29.19 (4.30)	21.80 (1.79)	29.15 (3.98)	1 vs. 2 *** 1 vs. 3 1 vs. 4 *** 2 vs. 3 *** 2 vs. 4 3 vs. 4 ***
**EOQ**	*F*(3, 1350) = 13.08, *p* < 0.001, *ƞ_p_*^2^ = 0.03				
Emotional overeating	4.65 (6.45)	4.83 (6.72)	6.10 (7.20)	8.31 (10.20)	1 vs. 2 1 vs. 3 * 1 vs. 4 *** 2 vs. 3 2 vs. 4 *** 3 vs. 4 ***
**EMS**	*F*(3, 1350) = 9.25, *p* < 0.001, *ƞ_p_*^2^ = 0.02				
Health	12.76 (4.39)	11.90 (4.72)	13.13 (4.52)	11.60 (4.29)	1 vs. 2 1 vs. 3 1 vs. 4 ** 2 vs. 3 * 2 vs. 4 3 vs. 4 ***
**EMS**	*F*(3, 1350) = 1.83, *p* > 0.05, *ƞ_p_*^2^ = 0.004				
Weight control	10.32 (4.24)	10.51 (4.42)	10.99 (4.51)	10.58 (4.10)	1 vs. 2 1 vs. 3 1 vs. 4 2 vs. 3 2 vs. 4 3 vs. 4
**EMS**	*F*(3, 1350) = 13.31, *p* < 0.001, *ƞ_p_*^2^ = 0.03				
Affect regulation	6.88 (3.64)	7.67 (4.45)	7.94 (4.15)	9.02 (4.94)	1 vs. 2 1 vs. 3 ** 1 vs. 4 *** 2 vs. 3 2 vs. 4 * 3 vs. 4 **
**MEQ**	*F*(3, 1350) = 8.01, *p* < 0.001, *ƞ_p_*^2^ = 0.02				
Recognition	26.95 (4.31)	25.92 (4.42)	26.43 (4.66)	25.26 (4.54)	1 vs. 2 1 vs. 3 1 vs. 4 *** 2 vs. 3 2 vs. 4 3 vs. 4 **
**MEQ**	*F*(3, 1350) = 4.52, *p* < 0.01, *ƞ_p_*^2^ = 0.01				
Awareness	30.92 (6.93)	29.33 (7.20)	31.46 (6.68)	30.27 (6.80)	1 vs. 2 1 vs. 3 1 vs. 4 2 vs. 3 ^**^ 2 vs. 4 3 vs. 4 ^⸸^

EOQ—the Emotional Overeating Questionnaire; EMS—the Eating Motivation Survey; MEQ—the Mindful Eating Questionnaire; healthy body weight: 18.5 ≥ BMI ≤ 24.99 kg/m^2^; overweight: BMI ≥ 25 kg/m^2^; COVID-related stress: “Yes” or “No”; CDRS—the Contour Drawing Rating Scale; * *p* < 0.05; ** *p* < 0.01; *** *p* < 0.001; ^⸸^ *p* = 0.076.

## Data Availability

The data that support the findings of this study are available from the corresponding author upon reasonable request.
